# Multidrug resistant and carbapenemase producing Enterobacteriaceae among patients with urinary tract infection at referral Hospital, Northwest Ethiopia

**DOI:** 10.1186/s13756-015-0054-7

**Published:** 2015-04-17

**Authors:** Setegn Eshetie, Chandrashekhar Unakal, Aschalew Gelaw, Birhanu Ayelign, Mengistu Endris, Feleke Moges

**Affiliations:** Department of Medicine, Debre Markos University, Debre Markos, Ethiopia; Department of Microbiology, University of Gondar, Gondar, Ethiopia; University of Gondar Hospital, Gondar, Ethiopia

**Keywords:** Carbapenemase, Enterobacteriaceae, Multidrug resistant, Urinary tract infection

## Abstract

**Background:**

Updates on the epidemiology of antibiotic resistance bacterial pathogens is important. This is because the spread of multidrug resistant enterobacteriaceae (MDRE) and recently carbapenemase producing enterobacteriaceae (CPE) have emerged as a major public health concern in patients with urinary tract infections (UTIs). This study is therefore, aimed to assess the prevalence and associated risk factors of MDR and CPE among patients with UTIs.

**Methods:**

A cross sectional study was conducted among 442 symptomatic UTI suspected patients. Data on socio-demographic characteristics, clinical information and possible risk factors were collected using structured questionnaire. Early morning mid-stream urine samples were collected and processed to characterize bacterial isolates. Disk diffusion method was used to determine the antibiotic susceptibility patterns of isolates. Carbapenemase producing strains were detected using CHROMagar KPC medium. Data were entered and analyzed using SPSS version 20. P-value <0.05 was considered as statistical significant.

**Results:**

Among 442 patients enrolled a total of 183 Enterobacteriaceae were recovered. Of these isolates; 160 (87.4%) were MDRE; the most common isolates were *K. pneumoniae* and *E.coli*. Five (2.73%) of the isolates were found to be carbapenemase producers and all of CPE strains were 100% ESBL producers. Significant drug resistances were observed among CPE compared to other MDRE, low resistance rates were noted to ciprofloxacin (20%). Being female (OR 4.46; P = 0.018), age (OR 1.08; P = 0.001), hospitalization (OR 5.23; P = 0.006), and prior antibiotic use (OR 3.98; P = 0.04) were associated risk factors for MDRE.

**Conclusion and recommendation:**

High rates of MDR (87.4%) were observed among enterobacteriaceae uropathogens; *K. pneumoniae* and *E.coli* were the principal MDR isolates. Overall prevalence of CPE was 2.73% and all of these strains were 100% ESBL producer. Attributing risk factors for MDR UTIs were found to be sex (female), age, hospitalization, and history of antibiotic therapy. Therefore, efforts should be made to reduce patient hospital stay and maximize rational use of drugs. Additional and vigorous investigation especially on CPE should be encouraged.

## Background

Urinary tract infections (UTIs) are one of the most common infectious diseases ranking next to upper respiratory tract infection. Urinary tract infections are often associated with significant morbidity and mortality. Worldwide, about 150 million people are diagnosed with UTI each year, costing the global economy in excess of 6 billion dollars [1]. In developing countries, including Ethiopia, the facilities for urine culture and antimicrobial susceptibility testing are still not sufficiently available, leading to improper diagnosis and irrational antibiotic treatment of UTI, which expedites the emergence of multidrug resistant (MDR) strains [2]. Gram negative bacteria, especially the family enterobacteriaceae are the common cause of both community and hospital acquired UTIs. *Escherichia coli* and *Klebsiella pneumoniae* are most commonly implicated among patients with UTI [3,4].

Previously, the emergences of MDR among enterobacteriaceae were mainly due to the production of enzymes, such as pencillinases, cephalosorinases, and extended spectrum β-lactamase (ESBL). However, recently carbapenemase production is one of the main mechanisms in the occurrence of drug resistance in the family of enterobacteriaceae. Carbapenemase producing enterobacteriaceae (CPE) are difficult to treat because they have high levels of resistance to antibiotics, which capable of break down all β-lactam agents including carbapenems and make it ineffective. Carbapenem such as imipenem, meropenem, ertapenem, & doripenem are considered as the last resort antibiotics to treat ESBL producing enterobacteriaceae [5,6].

Currently, increased burden of MDRE causing UTI compounded by harboring carbapenem resistance genes mainly among E*. coli* and *K. pneumonia.* These strains become a serious threat to public health, associated with high mortality rates and have the potential to spread widely. Infections are difficult, and in some cases impossible to treat and have been associated with mortality rates up to 50%. Due to the movement of patients throughout the health care system, if CPE is a problem in one facility, then typically they are a problem in other facilities in the region as well. Carbapenemase producing enterobacteriaceae are mostly endemic in specific geographical regions, but reports of their spread into other geographical locations are point of grave concern these days [5,7].

The aetiology of UTI and the antibiotic resistance of uropathogens have been changing over the past years, both in community and health care associated infections. Current knowledge on the burden and antimicrobial susceptibility pattern of the enterobacteriaceae isolates is essential for appropriate therapy, since those groups of bacteria are the main cause of UTIs and possess several mechanisms to dismantle currently available antibiotics including carbapenems and the condition in Ethiopia is not yet assessed. Therefore, the objective of this study was aimed to determine the prevalence and risk factors of MDR and CPE producing strains among patients with UTI at the University of Gondar Hospital, Ethiopia.

## Materials and methods

### Study area

The study was conducted at the University of Gondar Hospital. It is referral hospital that provides services to over 5 million inhabitants in the Northwest, Ethiopia. The hospital has an accredited referral level laboratory with 7 sections and a separate reception room. Microbiology section is one of the principal area, it is estimated that 9,600 samples delivered per annum to this working area. In this section, culturing is one of the main activities, mainly applicable for bacterial isolation and identification.

### Study design, participants and data collection

A cross-sectional study was conducted from February to May 2014. A total of 442 patients with symptomatic UTI were selected from both in and out-patients using systematic random sampling technique. Socio-demographic characteristics such as gender, age, residence, educational status and history of travel were gathered from eligible patients. Clinical features such as history of hospitalization, ICU admission, prior antibiotic use, prior UTI and chronic diseases, pregnancy status, presence of urinary catheter and mechanical ventilation were also collected. Moreover, after instructing how to collect urine specimen, about 20 ml of a clean catch morning mid-stream urine was collected from each patient [8] using a sterile screw-capped, wide-mouth container and labeled with the unique sample number, date and time of collection.

### Isolation and identification of enterobacteriaceae

Urine specimens were directly inoculated on 5% Sheep blood agar and incubated at 37°C for 24 hours. Urine culture was considered as positive, if it contains ≥10^5^ cfu/ml. Enterobacteriaceae from positive urine cultures were identified by their characteristic appearance on the media, gram staining reaction, by the pattern of biochemical profiles using standard procedures. Biochemical tests such as indole production, sugar fermentation, H_2_S and gas production, citrate utilization, motility test, urease test, oxidase, were used to identify enterobacteriaceae isolates [8].

### Susceptibility and carbapenemase testing

Antibiotic susceptibility was performed by employing Kirby Bauer disk diffusion method using Mueller Hinton agar (Oxoid) in accordance with the guidelines of clinical and laboratory standards institute [9]. Enterobacteriaceae were tested against the following antibiotic disks (Oxoid): cefotaxime (CTX; 30 μg), ceftriaxone (CTR; 30 μg), cefepime (CPM; 30 μg), ceftazidime (CAZ; 30 μg), cefpodoxime (CPD: 30 μg), ciprofloxacin (CIP; 5 μg), tetracycline (TE; 30 μg), chloramphenicol (C; 30 μg), amoxicillin-calvulanic acid (AMC; 30 μg), naldixic acid (NA; 30 μg), gentamycin (GEN; 10 μg), ampicillin (AMP; 10 μg) and trimethoprim-sulfamethoxazole (SXT; 25 μg). After incubation of plates at 37°C for 24 hours, diameters of zone of inhibition were measured. Bacteria classified as susceptible, intermediate and resistant strains according to the criteria of the clinical and laboratory standards institute [9].

After antimicrobial susceptibility testing, all MDRE (resistance to 2 or more classes of antibiotics) isolates were collected and sub-cultured on CHROMagar ^TM^ KPC agar to determine carbapenemase production. After overnight incubation (24 hr), carbapenemase producing isolates were assessed by visualizing colonies with typical coloring characteristics. Carbapenemase producing *E. coli* developed dark pink to reddish colony features, while other enterobacteriaceae isolates produced metallic blue colonies [10]. Those Carbapenemase producing colonies with metallic blue color were further identified following different classical biochemical tests (. Besides, all CPE strains were tested whether they are extended spectrum beta- lactamase (ESBL) producer or not using CHROM agar ESBL medium.

### Quality control

All materials, equipment and procedures were adequately controlled. Culture media were tested for sterility and performance. Pre-analytical, analytical and post-analytical stages of quality assurance that are incorporated in standard operating procedures of the microbiology laboratory were strictly followed. Standard strains of *E.coli*® ATCC 25922 (positive control) and *S. aureus* ATCC® 25923 (negative control) were used to control the performance of CHROMagar ^TM^ KPC medium and other media. To standardize the inoculum density of bacterial suspension for a susceptibility test, 0.5 McFarland standards was used [9,10].

### Ethical consideration

This study was approved by research and ethics committee of School of Biomedical Laboratory Sciences, University of Gondar, Ethiopia. Informed written consent was also obtained from patients and/or guardians after explaining the objective of the study. The laboratory results were communicated with the physicians for better management of the patients.

### Data analysis and interpretation

Data were collected, summarized and analyzed using SPSS version 20 software and results were presented through tables, pie charts and graphs. Associations were measured using chi-square test, binary logistic regression. P-values < 0.05 were considered as statistically significant.

## Results

### Socio-demographic characteristics

A total of 442 patients with symptomatic UTI were enrolled in this study to investigate prevalence and associated risk factors of MDRE and CPE. Majority of the participants were females 282 (63.8%). The mean age of patients was 37.05 ± 10.5 years, 86 (19.5%) of the patients were younger than 16 years, and 73 (16.5%) were older than 60 years. Two hundred fifty two (57.0%) of patients were rural residents, and majority, 286 (64.7%) of study participants had educational level of elementary school and below (Table 1).

**Table 1:** **Socio-demographic characteristics of UTI suspected patients at the University of Gondar Hospital, February to May 2014 (N = 442)**

**Variables**		**Frequency**	**Percentage**
Sex	Male	160	36.2
	Female	282	63.8
Age (Years)	≤15	86	19.5
	16-30	84	19
	31-45	101	22.9
	46-60	98	22.2
	≥61	73	16.3
Residence	Rural	252	57
	Urban	190	43
Educational status	Illiterate	196	44.3
	Primary school	90	20.4
	Secondary school	69	15.6
	Diploma and Above	87	19.7
Sender of the patient	Outpatient	212	48
	Inpatient	230	52

### Multidrug resistance and carbapenemase producing enterobacteriaceae in UTI suspected patients

Among study participants, 183 (41.4%) patients had positive urine culture with a single non-duplicate isolates of enterobacteriaceae. The most common isolates were *E.coli* 112 (61.2%) followed by *K. pneumoniae* 29 (15.8%) and *E. aerogenes* 13(7.1%) (Table 2). The isolates were tested for antimicrobial susceptibility, 160 (87.4%, 95% CI; 82–92.3%) of them showed resistance to two or more classes of antibiotics. Among MDR strains, only 1 (0.6%) isolate was resistant to 2 classes of antibiotics, the rest 159 (99.4%) were resistant to three or more classes of antibiotics. Result of drug resistance patterns compared within species showed that, 28 (95.6%) of *K. pneumoniae* and 104(92.9%) of *E. coli* were MDR isolates (Table 2).

**Table 2:** **Multidrug resistance pattern of enterobacteriaceae among UTI suspects at the University of Gondar Hospital, February to May 2014**

**Isolates**	**Degree of resistance**										**Total MDR isolates (≥R2)**
	**R0**	**R1**	**R2**	**R3**	**R4**	**R5**	**R6**	**R7**	**R8**	**≥R9**	
*E. coli* (N = 112)	2 (1.8)	6 (5.4)	^__^	5 ( 4.5)	28 (25)	24 (21.4)	32 (28.6)	9 (8.0)	4 (3.6)	2 (1.8)	104 (92.9)
*K. pneumoniae* (N = 29)	^__^	1 (3.4)	^__^	3 (10.3)	5 (17.2)	6 (20.7)	4 (13.8)	3 (10.3)	4 (13.8)	3 (10.3)	28 (95.6)
*Enterobacter* spp*.* (N = 16)	2 (12.5)	1 (6.3)	^__^	1 (6.3)	2 (12.5)	3 (18.8)	5 (31.3)	1 (6.3)	^__^	1 (6.3)	13 (81.3)
*Citrobacter* spp. (N = 6)	1 (16.7)	^__^	1 (16.7)	^__^	1 (16.7)	^__^	2 (33.3)	^__^	1 (16.7)	^__^	5 (83.3)
*Proteus* spp. (N = 9)	5 (55.6)	3 (33.3)	^__^	^___^	^___^	1 (11.1)	^___^	^__^	^__^	^__^	1 (11.1)
Other *Klebsiella* spp. (N = 11)	__	2 (18.2)	^__^	^__^	^___^	3 (27.3)	3 (27.3)	1 (9.1)	1 (9.1)	1 (9.1)	9 (81.8)
Total (N = 183)	10 (5.5)	13 (7.1)	1 (0.5)	9 (4.9)	36 (19.7)	37 (20.2)	46 (25.1)	14 (7.7)	10 (5.5)	2 (1.1)	160 (87.4)

Note: Data are in number (%) unless otherwise indicated.

R0: susceptible to all antibiotics, R1-8: resistance to 2, 3, 4, 5, 6, 7, and 8 antibiotics, ≥R9: resistance to 9 or more antibiotics, ≥R2: resistance to 2 or more antibiotics.

Of the 183 enterobacteriaceae isolates, 160 (87.4%) were MDR strains, and these strains were tested for carbapenemase production by using phenotypic methods (CHROMagar KPC media). A total of 5 bacterial strains were found to be CPE producers, notably *E.coli* (2), *K. pneumoniae* (2) and *E. aerogenes* (1). All of the isolates were from hospital admitted patients. The overall prevalence of CPE was 2.73% (95%CI; 0.5-5.5%) among all isolates and 3.1% among MDRE isolates. Besides, all CPE strains were 100% ESBL producer, which were demonstrated by using phenotypic methods (CHROMagar ESBL media).

### Rate of resistance for different antibiotics tested in MDRE and CPE Isolates

The overall resistance profile of MDRE isolates are shown in Table 3. High resistance rate were observed to ampicillin (97.5%) followed by cotrimoxazole (64.4%), and chloramphenicol (61.2%). Whereas, ciprofloxacin, cefepime, and ceftriaxone had an overall resistance rates of 2.5%, 10.6%, and 11.9%, respectively. Species specific antibiotic resistance rates revealed that more than 55% of *E.coli* isolates were resistant to ceftazidime, gentamycin, chloramphenicol, cotrimoxazole, and ampicillin and low rates of resistance were observed in ciprofloxacin (1%), cefepime (8.7%) and ceftriaxone (11.5%). Over 60% of *K. pneumoniae* were exhibited resistance to amoxicillin-calvulanic acid, chloramphenicol, cefpodoxime, and ampicillin, relatively low resistance rates were observed to ciprofloxacin (10.7%), cefepime (14.3%), and ceftriaxone (17.9%).

**Table 3:** **Antibiotic resistance patterns of MDRE among study participants: University of Gondar Hospital, February to May 2014**

**MDR isolates**	**Antibiotics**												
	**CTX**	**CAZ**	**CTR**	**CPD**	**CPM**	**CIP**	**TE**	**SXT**	**C**	**AMP**	**NA**	**GEN**	**AMC**
*E.coli* (N = 104)	25 (24.0)	58 (55.8)	12 (11.5)	43 (41.3)	9 (8.7)	1 (1)	49 (47.1)	72 (69.2)	61 (58.7)	103 (99)	19 (18.3)	59 (56.7)	47 (45.2)
*K. pneumoniae* (N = 28)	8 (28.6)	16 (57.1)	5 (17.9)	18 (64.3)	4 (14.3)	3 (10.7)	15 (53.6)	14 (50)	18 (64.3)	26 (92.9)	7 (25)	16 (57.1)	17 60.7)
K. ozaenae (N = 6)	1 (16.7)	4 (66.7)	0	3 (50)	0	0	4 (66.7)	5 (83.3)	5 (83.3)	6 (100)	2 (33.3)	4 (66.7)	5 (83.3)
*E. aerogenes* (N = 12)	4 (33.3)	7 (58.3)	2 (16.7)	5 (41.7)	3 (25)	0	5 (41.7)	5 (41.7)	7 (58.3)	12 (100)	2 (16.7)	7 (58.3)	9 (75)
*Citrobacter* spp (N = 5)	1 (20)	2 (40)	0	1 (20)	0	0	4 (80)	3 (60)	4 (80)	4 (80)	1 (20)	4 (80)	2 (40)
*Others (N = 5)	2 (40)	3 (60)	0	1(20)	1(20)	0	2(40)	4 (80)	3(60)	5(100)	1(20)	4(80)	1(20)
Total MDRE N = 160 (87.4%)	40 (25)	90 (56.2)	19 (11.9)	71 (44.4)	17 (10.6)	4 (2.5)	79 (49.4)	103 (64.4)	98 (61.2)	156 (97.5)	32 (20)	94 (58.8)	81 (50.6)

Note: Data are in number (%) unless otherwise indicated. *Others = K. oxytoca (N = 3); *E. cloacae* (N = 1); *P. vulgaris* (N = 1).

CTX: Cefotaxime, CAZ: Ceftazidime, CTR: Ceftriaxone, CPD: Cefpodoxime, CPM: Cefepime, CIP: Ciprofloxacin, TE: Tetracycline, SXT: Cotrimoxazole, C: Chloramphenicol, NA: Naldixic acid, GEN: Gentamycin, AMC: Amoxicillin-Calvulanic acid.

**Figure 1:**
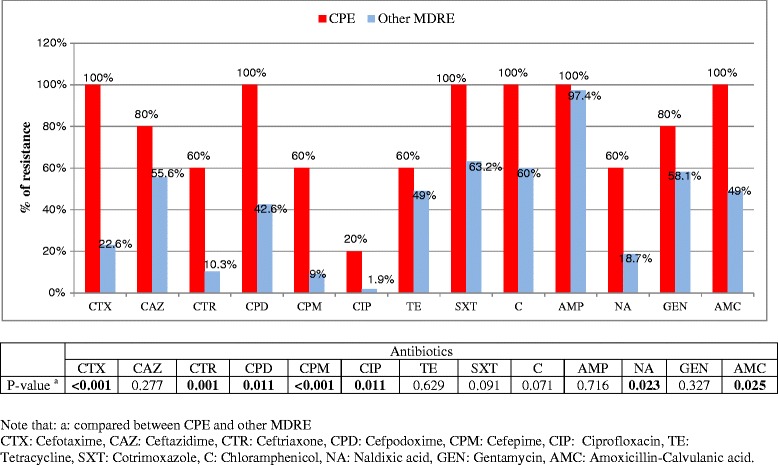
Antibiotic resistance rate of CPE isolates compared to other MDRE among study participants: University of Gondar Hospital, February to May 2014.

The overall resistance pattern of CPE isolates are summarized in Figure 1. All isolates were 100% resistant to cefotaxime, cefpodoxime, cotrimoxazole, chloramphenicol, ampicillin, and amoxicillin-calvulanic acid. However, only 20% of strains were resistant to ciprofloxacin. The overall antibiotic resistance rates of CPE isolates were significantly higher than other MDRE strains for more than half of tested antibiotics including cefotaxime (100% versus 22.6%; P < 0.001), ceftriaxone (60% versus 10.3%; P = 0.001), cefpodoxime (100% versus 42.6%; P = 0.011). On the other hand the difference in antibiotic resistance rate of CPE to ceftazidime, tetracycline, cotrimoxazole, chloramphenicol, ampicillin, and gentamycin were not statistically significant compared to other MDRE isolates.

### Risk factors for MDRE and CPE among study participants

Risk factors associated with MDRE UTIs were analyzed by comparing patients with and without MDRE UTIs. Bivariate analysis showed that, age, hospitalization for the last 12 months, prior urinary tract infection for the past 12 months, prior antibiotic use for the past 6 months were associated with MDRE infections. In the analysis of multivariate logistic regression, independent risk factors for MDRE were prior antibiotic use, and hospitalization since the past 12 months, age, and sex (female) (Table 4).

**Table 4:** **Risk factors associated with MDRE among UTI suspected patients at the University of Gondar Hospital, February to May 2014**

**Risk factors**	**MDRE**		**Bivariate analysis**		**Multivariable analysis**	
	**Yes (N = 160)**	**No (N = 23)**	**COR (95% CI)**	**P-value**	**AOR (95% CI)**	**P-value**
**Sex**						
Female	106	12	1.79 (0.75 – 4.34)	0.191	4.46 (1.29 – 15.35)	**0.018**
Male	54	11	1		1	
**Age (years)**						
Mean age	38.5	13.2	1.08 (1.05 - 1.12)	**<0.001**	1.08 (1.03 – 1.13)	**0.001**
**Hospitalization**						
Yes	114	9	3.86 (1.56 – 9.53)	**0.003**	5.22 (1.59 – 17.17)	**0.006**
No	46	14	1		1	
**Prior UTI**						
Yes	65	4	3.25 (1.06 – 9.99)	**0.040**	2.41 (0.56 – 10.34)	0.239
No	95	19	1		1	
**Prior antibiotic use**						
Yes	129	7	9.51 (3.60 – 25.11)	**< 0.001**	3.98 (1.056 – 14.97)	**0.041**
No	31	16	1		1	

Note that: COR: crude odds ratio, AOR: adjusted odds ratio, CI: confidence interval.

## Discussion

The overall prevalence of MDR among enterobacteriaceae isolates identified from patients with symptomatic UTI was 87.4% (95% CI; 82–92.3%), which is similar with the results from previous study in Gondar (85.5%) and Mozambique (88.2%) [11,12] while it was higher than reports from other study in Ethiopia: Gondar (68%), and Dessie (74.6%) [13,14] and many other countries, such as USA (19.1%), Belgium (62%), and Italy (62%), Nepal (40.1%, 64.04%) [15-19]. However, it was lower than reports from different parts of Ethiopia such as Gondar (93.5%), Bahirdar (95.6%), and Jimma (100%) [20-22]. The variation in prevalence of MDRE isolates could be due to increase trend of MDR strains with time, difference in study period and study population.

The present study showed that, *K. pneumoniae* (95.6%) and *E.coli* (92.9%) were found to be the principal MDR isolates. Although the rate of proportion of MDR is different in different area similar group of bacteria were reported in Bahirdar, Ethiopia *E.coli* (94.6%) & *K. pneumoniae* (80%) [21] and Nepal, *E.coli* (74%) and *K. pneumoniae* (44%) and Dakar, *E. coli* and *K. pneumoniae* (89%) [23] were the predominant MDR uropathogens [19]. These pathogens are the most common isolates in both hospital and community acquired urinary tract infections. Besides, these bacteria are frequently difficult to treat because of both their intrinsic and acquired resistance to multiple groups of antimicrobial agents [3,4].

Among 183 enterobacteriaceae isolates, 5(2.73%) were found to be carbapenemase producers. Comparable result were reported in studies from Morocco (2.8%) [24], Bangladesh (4.8%) [25], Taiwan (2.5%) [26], Belgium (3.5%) [27], and India (5.4%) [28]. However, this was lower than from studies in Pakistan (8.6%) [29], Turkey (10.9%) [30], India (12.9%) [31], Nigeria (14%, 33.5%) [32,33], Iran (14.5%) [34], and USA (21%) [35]. The difference in the prevalence of CPE in different studies may be due to trends in the utilization of carbapenems and other broad spectrum antibiotics, cultural/traditional relationships, cross boarder transfer of patients with other countries of high prevalence. Additionally, difference in target population, sample size and methodological variability could bring variation in the epidemiology of CPE.

Moreover, according to World Health Organization (WHO) 2014 report [36], the epidemiology of CPE has not well studied in developing countries, therefore the report insisted that integrated surveillance program and involvement of very active investigation have to be maximized in order to know the extent of resistant strains in these countries. Even though carbapenems drugs are not formally introduced in to Ethiopia, as the report claimed that increase international travel, globalization and migration might have contributing role in the dissemination of resistant strains from potentially risk countries [36]. Especially, in this study area; there is high tourist flow, and many of residences have relatives from abroad, which may have an impact on the emergence of carbapenemase producing strains in this locality, particularly.

All carbapenemase producing isolates were from hospital admitted patients. This was supported by the fact that inpatients admitted to critical care units for treatment of acute emergencies and chronic diseases are especially liable to get CRE infections because of the presence of highly resistant organisms available in an environment and selective pressure on them due to overuse of antibiotics [37].

In the present study *K. pneumoniae*, *E.coli,* and E*. aerogenes* were carbapenemase producers. This result was supported by the reports from European Antimicrobial Resistance Surveillance Network (EARS-Net) that many of carbapenemase producers were *K. pneumoniae* followed by *E.coli* and *Enterobacter* spp., [38]. The same situations were also notified in finding from Turkey and Morocco indicated that *K. pneumoniae* were the principal isolate followed by *E.coli* and K*. oxytoca* [30,35]. On the other hand, a study from Nigeria demonstrated that *E.coli* was the main carbapenemase producer followed by *Proteus* spp. and *K. pneumoniae* [32]. The variation among studies with regard to the proportion of carbapenemase producing isolates; could be due to difference in geographical distribution of isolates, target population, sample size, and methodology used in each investigation.

In Bivariate analysis, age (years), hospitalization within the past 12 months, prior antibiotic therapy in the past 6 months, and prior UTI in the past 12 months were associated with MDRE UTI in this study. Likewise, in multivariable analysis, age, being female, hospitalization within the past 12 months, and prior antibiotic use in the past 6 months were the independent risk factors for MDRE UTIs. The same result was documented in a study done from USA [15]. However, additional risk factors like health care associated risks (use of urinary catheter, mechanical ventilation, and hemodialysis) were identified in the former study, which were not indicated in this study.

## Conclusion and recommendation

High rates of multi-drug resistance were observed among enterobacteriaceae uropathogens, (87.4%). Very high resistance was reported to ampicillin, followed by cotrimoxazole and chloramphenicol. Isolates of *K. pneumonia* and E*. coli* were the principal MDR isolates. Overall prevalence of CPE was 2.73% and all CPE strains were 100% ESBL producer and completely resistant to ampicillin, cefotaxime, cefpodoxime, cotrimoxazole, chloramphenicol, and amoxicillin-calvulanic acid. The only drug that shows low resistance rate was ciprofloxacin. Being female, age, hospitalization, and prior antibiotic use were associated risk factors for MDRE. Therefore, efforts should be made to reduce patient hospital stay and maximize rational use of drugs. Additional and vigorous investigation especially on CPE should be encouraged.

## Abbreviations

CPE: Carbapenemase producing enterobacteriaceae
ESBL: Extended spectrum β-Lactamase
ICU: Intensive Care Unit
IMP: Imipenemase
KPC: *Klebsiella pneumoniae* Carbapenemase
MDR: Multi-drug resistant
MDRE: Multi-drug resistant enterobacteriaceae
NDM- 1: New Delhi Metallo-beta-lactamase
OXA-48: Oxacillin-hydrolzying metallo-β-lactamases
UTI: Urinary tract infection
VIM: Verona integron encoded Metallo-beta-lactamase
